# 19. Unusual findings in giant cell arteritis

**DOI:** 10.1093/rap/rky033.011

**Published:** 2018-09-20

**Authors:** Hannah Zacharias, Kieran Sandhu, Malgorzata Magliano

**Affiliations:** Rheumatology, Stoke Manderville Hosital, Aylesbury, UNITED KINGDOM

**Introduction:** The classic presentation of giant cell arteritis (GCA) is captured by the 1990 American College of Rheumatology criteria, which require fulﬁllment of 3 out of 5 core features; age of 50 years or older at onset, a new headache, a clinical temporal artery abnormality, elevated erythrocyte sedimentation rate (ESR) of at least 50 millimeter/hour, and an abnormal temporal artery biopsy (TAB). Neuro-ophthalmologic complications are well recognised and unfortunately despite effective therapy remain relatively common reported in 15 to 20 percent of patients with GCA. Blindness is the most feared complication of GCA and occurs most often secondary to anterior ischemic optic neuropathy, posterior ciliary arteritis is the most common pathology. Visual loss may be proceeded by amaurosis fugax, diplopia, and/or vision loss in the other eye, notably second eye involvement can occur within hours to days in untreated patients. Diplopia occurs in approximately 5% of patients, secondary to ischemic damage to the oculomotor system, including the brainstem, oculomotor nerves, and the extraocular muscles themselves and may be accompanied by ptosis and miosis, appearing as a Horner syndrome. In the context of other symptoms suggestive of GCA, diplopia has a high specificity for the disease and can be a predictor of visual loss. We describe the case of a patient with acute diplopia consistent with six nerve palsy and temporal discomfort as presenting signs for a differential diagnosis of GCA.

**Case description:** We present an 88 year old lady with six day history of sudden onset of diplopia. In the preceeding four weeks she complained of right temporal headache and temporal ad facial tenderness. There was no history of jaw claudication or other visual disturbance. She had poor appetite, but no night sweats or fevers. She reported dry mouth, painful thighs and difficulties walking but no neck or shoulder symptoms. On examination temporal arteries were non-tender and pulsatile. Blood pressure was 122/65, heart rate was regular, there was very soft ejection systolic murmur heard over the aorta. There were no bruits and peripheral pulses were normal. She had right six nerve palsy, fundoscopy was normal. Bloods revealed a raised ESR 78 mm/hr, with normal CRP 2.2 mg/L WCC 8.0109/L. CT head was normal. She was commenced on prednisolone 60 mg for likely giant cell arteritis. Three years previously she had an asymptomatic skin coloured lesion with raised edges excised from the right nasal region. Histology showed a completely excised, poor differentiated squamous cell carcinoma with 4 mm margins. No lymphadenopathy. One year after the excision she was noted to have swelling of right cheek, thought to be lymphoedema post surgery. It was also noted that she had numbness over the area of surgery. Shortly after starting steroids the patient reported worsening discomfort and swelling in the area and she underwent incision and drainage of the swelling which revealed a localised abscess from a retained stitch from SCC excision two years prior. Microscopy and culture were negative, histology not performed and she was treated with a course of antibiotics. Temporal artery biopsy showed no evidence of inflammation. Her symptoms of pain affecting the right forehead and maxillary area were unimproved, the sixth nerve palsy was unchanged and there was no systemic symptoms. Repeat inflammatory markers, have normalised showing ESR of 9 mm/hr and CRP <1 mg/L. Due to ongoing pain she had an MRI which revealed recurrence of right nasal alar squamous cell carcinoma with perineural evasion along the infraorbital nerve and into the orbital apex. Neurological examination at this point showed reduced V2 sensation, anaesthetic cornea causing a large neurotrophic corneal ulcer in addition to persistent right VI cranial nerve palsy. The patient was at risk of corneal perforation and was treated with hourly lubricants and chloramphenicol ointment. She was then given botulinum toxin injection to the levator to induce a ptosis. Following her MRI scan, the case was discussed at the skin MDT however due to increasing frailty the decision for palliation was made. Her neuropathic symptoms were managed with gabapentin.

**Discussion:** The diagnosis of CGA is based on the history, the clinical picture, and the temporal artery biopsy. Involvement of cranial nerves, leading to diplopia is a known complication of CGA and as previously stated in the context of other symptoms suggestive of GCA, diplopia has a high specificity for the disease. This patient fulfilled criteria for age, new headache and raised ESR based on this a diagnosis of GCA appeared likely. Improvement of inflammatory markers could have be attributed to either therapeutic steroids or incision and drainage with antibiotic course. High clinical suspcicion was therefore required in the context of previous history of malignancy when the patient reported poor clinical response.

**Key Learning Points:** This case highlights that patients presenting with neurological disease of possible vascular origin warrant an ESR, consideration of temporal artery biopsy and perhaps a therapeutic trial of steroids but in the absence of a response alternative diagnosis should be considered.

**Disclosure: H. Zacharias:** None. **K. Sandhu:** None. **M. Magliano:** None.

